# Meeting the challenges of NTM-PD from the perspective of the organism and the disease process: innovations in drug development and delivery

**DOI:** 10.1186/s12931-022-02299-w

**Published:** 2022-12-24

**Authors:** Roald van der Laan, Andy Snabilié, Marko Obradovic

**Affiliations:** 1 Insmed B.V., Utrecht, The Netherlands; 2 Insmed Germany GmbH, Frankfurt Am Main, Germany

**Keywords:** Non-tuberculous mycobacteria, NTM, NTM pulmonary disease, NTM lung disease, Amikacin, Liposome, ALIS

## Abstract

Non-tuberculous mycobacterial pulmonary disease (NTM-PD) poses a substantial patient, healthcare, and economic burden. Managing NTM-PD remains challenging, and factors contributing to this include morphological, species, and patient characteristics as well as the treatment itself. This narrative review focusses on the challenges of NTM-PD from the perspective of the organism and the disease process. Morphological characteristics of non-tuberculous mycobacteria (NTM), antimicrobial resistance mechanisms, and an ability to evade host defences reduce NTM susceptibility to many antibiotics. Resistance to antibiotics, particularly macrolides, is of concern, and is associated with high mortality rates in patients with NTM-PD. New therapies are desperately needed to overcome these hurdles and improve treatment outcomes in NTM-PD. Amikacin liposome inhalation suspension (ALIS) is the first therapy specifically developed to treat refractory NTM-PD caused by *Mycobacterium avium* complex (MAC) and is approved in the US, EU and Japan. It provides targeted delivery to the lung and effective penetration of macrophages and biofilms and has demonstrated efficacy in treating refractory MAC pulmonary disease (MAC-PD) in the Phase III CONVERT study. Several other therapies are currently being developed including vaccination, bacteriophage therapy, and optimising host defences. Newly developed antibiotics have shown potential activity against NTM-PD and include benzimidazole, delamanid, and pretomanid. Antibiotics commonly used to treat other infections have also been repurposed for NTM-PD, including clofazimine and bedaquiline. Data from larger-scale studies are needed to determine the potential of many of these therapies for treating NTM-PD.

## Background

Non-tuberculous mycobacterial pulmonary disease (NTM-PD) is a difficult-to-treat condition that is increasing in prevalence globally and presents a substantial burden to patients [1]. NTM-PD can have a significant impact on patients, causing lung function decline, worsening comorbidities, and reduced health-related quality of life and social functioning compared with the general population [2–10]. All-cause mortality in patients with NTM-PD can be up to four times higher than the general population, independent of other factors [8, 11–13]. NTM-PD is also associated with substantial economic burden, significantly greater risk of all-cause hospitalisation, and increased healthcare expenditure [13–15].

Many factors contribute to the challenges of treating NTM-PD; these include characteristics of the non-tuberculous mycobacteria (NTM) species and its intrinsic resistance capabilities [16, 17] as well as the ability of NTM to evade host defences through sequestration in biofilms and macrophages in the lung, making effective antibiotic penetration and treatment difficult [18]. In addition, symptoms of NTM-PD are non-specific and mirror those of underlying conditions, and diagnosis is often delayed for a number of years for some patients who have moderate-to-severe symptoms at the time of diagnosis [19–21]. The decision to treat is challenging and depends on the severity of disease, causative NTM species, and the patient’s goals [22]. Treatment is also lengthy, typically lasting for more than 12 months with multidrug regimens [19, 22].

The objective of this narrative review is to outline many of these factors and their implications for the treatment of NTM-PD, specifically focusing on challenges from the perspective of the NTM organism and disease process, and to discuss new treatment approaches already available or in development that aim to overcome these challenges.

## Methods

We conducted a narrative review of literature retrieved from PubMed. The authors selected publications related to NTM based on title and abstract, published between 1990 and 2021. Relevant information was also retrieved from clinicaltrials.gov. Each publication was reviewed subjectively, and publications considered most relevant or robust were included in this narrative review.

### Overview of the challenges of NTM infection—species virulence, at-risk patients, and treatment outcomes

The prevalence of NTM-PD is increasing globally [23], with recent reports estimating a prevalence of 2.3–6.5 per 100,000 in Europe [24–26]. In Japan, prevalence rates are even higher at an estimated 33–65 cases per 100,000 [27], and incidence rates in the United States of 3.1 per 100,000 in 2008 increasing to 4.7 per 100,000 in 2015 [28]. Predictive modelling studies using machine learning with United Kingdom and German databases have not only revealed an increase in NTM-PD prevalence but also a higher prevalence of potentially undiagnosed patients [29, 30].

**Fig. 1:**
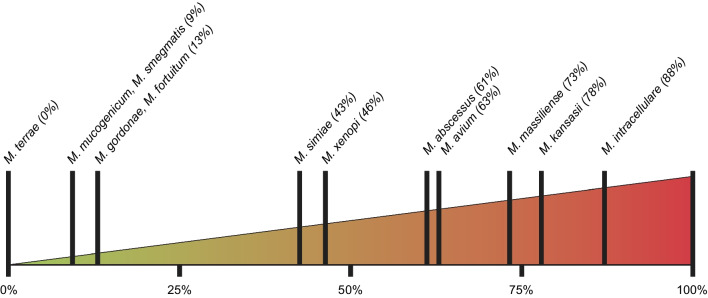
Clinical relevance of non-tuberculous mycobacteria species [31]

Despite the ubiquitous nature of NTM in the environment, exposure does not equate to infection and NTM-PD remains a rare disease. The clinical relevance of mycobacterial species and their ability to cause disease differs, with the most clinically relevant species being *Mycobacterium avium* complex (MAC) (e.g., *M. intracellulare, M. avium* and *M. chimaera), M. kansasii,* and *M. abscessus* complex (*M. abscessus* subsp. *abscessus, M. abscessus* subsp. *massiliense* and *M. abscessus* subsp. *bolletii*) [31–33] (Fig. 1).

It is the interplay of factors of host susceptibility, NTM species virulence, and environmental exposure that determine the disease trajectory. Host susceptibility factors including underlying lung conditions, immunosuppression, and a selection of morphological patient characteristics are shown in Table 1 [23, 34–38]. Frequent exposure to environmental sources of NTM such as household water, soil, and bathrooms can also increase risk of infection, and reinfection from these sources is common [39, 40].

**Table 1:** Predisposing risk factors for non-tuberculous mycobacterial pulmonary disease [23, 35, 41–44]

Study description	Relative risk, odds ratio or relative prevalence
Bronchiectasis	44.0–187.5
History of TB	178.3
Low bodyweight	9.1^a^
Thoracic skeletal abnormalities	5.4
Lung cancer (neoplasms of larynx, trachea, and bronchus)	3.4
Immunomodulatory drugs/anti-TNF agents	1.3 (undefined) 2.2 (anti-TNF agents)
Chronic obstructive pulmonary disease	2.0–10.0
Steroid use	1.6–8.0
Rheumatoid arthritis	1.5–1.9^b^
Gastroesophageal reflux disease	1.5^a^–5.3^b^

a. Estimated from published data. b. Hazard ratio, fully adjusted for age, sex, income, rurality, and comorbidities for non-tuberculous mycobacteria (HIV, chronic obstructive pulmonary disease and gastroesophageal reflux disease). TB, tuberculosis; TNF, tumour necrosis factor. Adapted from [23]

**Fig. 2:**
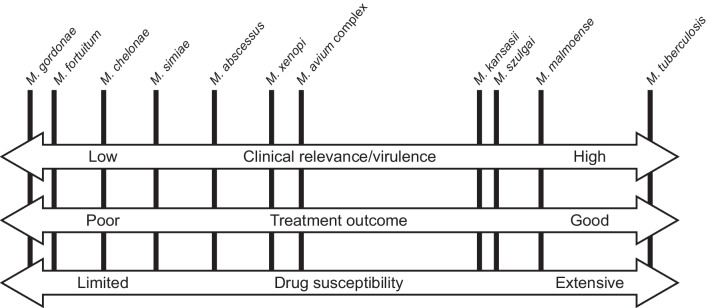
Relationship between the virulence of non-tuberculous mycobacteria species, treatment outcomes, and drug susceptibility [42]

Treatment goals for NTM-PD are to improve clinical, radiologic, and microbiological aspects of the disease and to achieve sputum culture conversion [7, 22, 45, 46]. Treatment outcomes for NTM-PD are intimately linked with infecting NTM species (Fig. 2) [46], and treatment recommendations for the most clinically relevant species causing NTM-PD—MAC, *M. abscessus*, *M. xenopi*, and *M. kansasii*—are provided in the 2020 guidelines [22]. A major challenge in treating NTM-PD is the high level of treatment failure, which can range from approximately 25% to almost 60% depending on the NTM species [7, 9, 47], and in macrolide-resistant NTM-PD potentially more than 70% [48]. Treatment failure can also increase the risk of further lung damage, reduce quality of life, and increase mortality [1, 48, 49]. Treatment itself is also challenging, with the need for extended treatment duration of 12 months post-culture conversion for some species of NTM [22].

### Overview of the challenges of NTM organisms—biology, structure, and antibiotic resistance

**Fig. 3:**
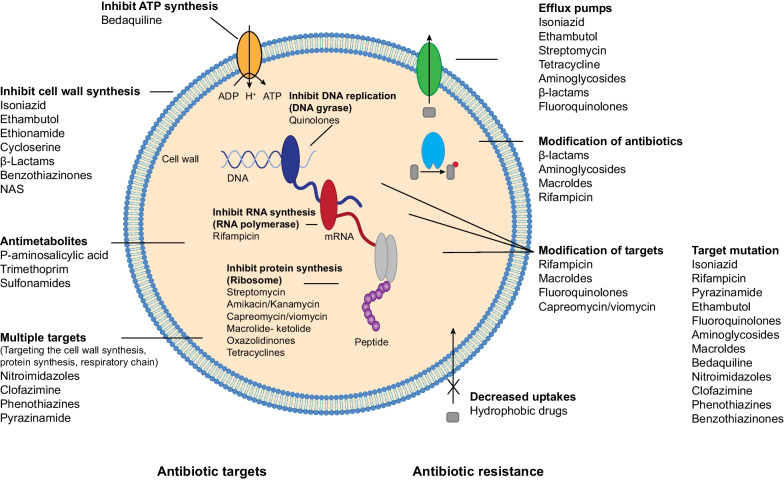
Mycobacterial resistance mechanisms to antibiotics used against non-tuberculous mycobacteria [17]

The life cycle and morphological characteristics of NTM bacteria create challenges for treatment as they exist as planktonic bacteria, can form biofilms, and invade eukaryotic cells [50, 51]. NTM are characterised by thick, hydrophobic cell walls, an ability to evade host defences through sequestration in and manipulation of macrophages, and an array of antimicrobial resistance mechanisms (Table 2; Fig. 3).

**Table 2:** Considerations and challenges to overcome in developing drugs to treat non-tuberculous mycobacterial pulmonary disease

Challenge	Detailed overview
NTM organism—hydrophobicity and innate resistance	• Permeability barrier because of hydrophobic, lipid-rich double membrane cell envelope • Prevention of antibiotic binding due to non-polar cell surface • Ability to switch morphology reversibly, which can vary drug susceptibility • Potential to express efflux pumps to prevent intracellular drug accumulation and enzymes to limit drug activity • Natural and acquired drug resistance through target gene polymorphisms to prevent drug binding and modification of target binding site upon drug exposure
Acquired drug resistance	• Genomic mutations (mutations in the target or other related genes to confer high-level resistance after long-course treatment) • Lateral gene transfer of drug resistance genes (less frequent but possible)
Correlation between in vitro MIC and clinical outcomes	• In vitro conditions to determine mycobacterial growth do not mimic the lung environment • Growth in airway mucous and biofilms
Intracellular growth and sequestration into phagocytic cells	• Intracellular growth, survival, and persistence (macrophages, monocytes) • Ability to escape from normal macrophage apoptosis mechanisms • Ability to limit normal acidification of phagolysosomes • Ability to decrease normal apoptosis mechanisms and block autophagy
Mucous and biofilm growth	• Ability to form and reside within biofilms • Capability of long-term viability due to ability to adopt a non-replicating dormant state under nutrient or oxygen starvation • High mucous production in NTM-PD assists in bacterial evasion from antimicrobial therapy and reduced antimicrobial susceptibility

MIC, minimum inhibitory concentration; NTM, non-tuberculous mycobacteria; NTM-PD, non-tuberculous mycobacterial pulmonary disease. Adapted from [52]

NTM are non-motile, rod-shaped, aerobic Gram-positive bacilli, with specific physiological characteristics such as long-chain mycolic acids in their cell wall that make NTM extremely hydrophobic and impenetrable [16]. Because of these characteristic cell wall features, NTM are intrinsically resistant to many antibiotics, making penetration into the bacteria extremely difficult, and those reaching the bacterial cell may be subject to efflux pumps or metabolising processes that modify either the antibiotic itself or its target [17] (Fig. 3). In addition, some species of NTM may harbour inducible resistance by activating certain genes upon exposure to antibiotics and can also acquire genetic mutations responsible for antibiotic resistance [17].

NTM are ubiquitous in the environment, rendering avoidance impossible [53]. Typically, NTM infection arises from inhalation of contaminated environmental particles such as aerosols and dust or aspiration of contaminated substances [18, 40, 50]. In the environment, biofilms containing NTM can be found in water distribution systems, while examples of intracellular niches include amoeba in water [54, 55].

In infected individuals, NTM can form biofilms on the alveolar wall and invade cells including epithelial cells and alveolar macrophages [54, 55]. Alveolar macrophages are believed to be the main reservoir of NTM in NTM-PD [54, 55]. Once inside alveolar macrophages, NTM augment macrophage functions including cytokine production and release, as well as phagosome–lysosome fusion inhibition. This allows bacteria to survive and replicate intracellularly before macrophages undergo apoptosis, releasing the bacteria to infect neighbouring macrophages and triggering a proinflammatory response [34, 56–60]. Antibiotic penetration of intracellular spaces is variable, with some antibiotics such as macrolides able to penetrate macrophages and biofilms whereas others, such as amikacin, poorly penetrate thereby limiting access to bacteria and effectively reducing their bactericidal potential despite most NTM being susceptible [61–64].

The life cycle of NTM bacteria contributes to a reduced susceptibility to antibiotics. Under conditions of nutrient starvation *M. intracellulare* and *M. avium* demonstrate a biphasic approach: an adaptive phase lasting around one week when bacterial viability plummets by 50% followed by a metabolically dormant phase—the persistence phase [65]. In these two phases, upregulation of genes and accumulation of proteins drive antibiotic susceptibility decline as changes in lipid metabolism gain traction, reducing cell wall permeability beyond that afforded by the cell wall to reduce antibiotic permeability, rendering bacteria ‘tolerant’ to antibiotics [65]. In in vitro biofilms, extracellular DNA has been shown to be integral to the structural integrity of *M. avium* subsp. *hominissuis*, increasing tolerance to antibiotics [65]. Additionally, upregulation of expression of efflux pumps contributes to reduced antibiotic susceptibility and a study of efflux pump inhibitors (such as verapamil) has demonstrated increased antibiotic susceptibility [66] suggesting that efflux pump inhibitors could, potentially, provide adjunctive therapeutic support to target intracellular and extracellular antibiotic-tolerant mycobacteria. *M. avium* contains phosphate-sensing genes, that are comparable with those in *M. tuberculosis* [65]. In *M. tuberculosis*, phosphate sensing, which is upregulated during phases of nutrient starvation, is an important mechanism that provides organisms with antibiotic tolerance [67]. Whether gene homology in other mycobacteria confers similar tolerance effects is yet unknown and further studies are required.

Antibiotic resistance is a key concern in the treatment of NTM-PD, as patients with resistant disease have poor culture conversion rates and high 5-year mortality rates [48, 49, 68]. Resistance to macrolides is of particular concern as this forms the backbone therapy for NTM-PD caused by MAC and *M. abscessus*, and acts as an alternative therapy to isoniazid in *M. kansasii*-PD and moxifloxacin in *M. xenopi*-PD [22]. In MAC, macrolide resistance can result from modifications of drug binding sites through mutations in the 23S rRNA gene that prevent macrolides binding to ribosomes [69].

Prophylactic macrolide therapy and macrolide monotherapy in the presence of NTM infection are risk factors for macrolide resistance [69] and recent guidelines for bronchiectasis recommend testing for, and excluding, NTM before long-term macrolide therapy is put in place for exacerbations [70]. In *M. abscessus*, macrolide resistance can be intrinsic owing to the presence of the ribosomal methyltransferase gene *erm*(41). *Erm*(41) can also be induced to provide resistance to macrolides over time, whereas in *M. kansasii*, resistance to rifampicin can be acquired via mutations in the gene coding for RNA polymerase [69].

NTM guidelines recommend susceptibility testing before initiating regimens with drugs for which there are clear correlations between in vitro activity and treatment outcomes, such as macrolides and amikacin for MAC and *M. abscessus*, and rifampicin for *M. kansasii* [22]. However, differences in growth conditions for NTM in vitro and in the lung environment can result in a poor correlation between minimum inhibitory concentration (MIC) and clinical outcomes [52].

To be effective, antimicrobial treatment must overcome all these challenges to reach bacteria and facilitate eradication (Fig. 2). Development of new therapies for NTM-PD need to consider these multiple hurdles provided by NTM organisms (Table 2) [52].

### Meeting the challenges of NTM-PD

International 2020 guidelines outline therapeutic options for four of the most common NTM species that cause pulmonary disease: MAC, *M. abscessus*, *M. xenopi* and *M. kansasii* (Table 3) [22]. Although, due to relatively high rates of treatment failure, development of further treatment options for NTM-PD are a priority. Treatment options include new therapeutic delivery approaches or new therapies to treat NTM-PD [71].

**Table 3:** Overview of guideline-based therapy for pulmonary disease caused by common NTM pathogens

Organism		Number of drugs	Preferred drug regimen	Dosing frequency
MAC	Nodular-bronchiectatic disease	3	Macrolide Rifampicin Ethambutol	3 times weekly
	Cavitary disease	≥ 3	Macrolide Rifampicin Ethambutol Amikacin IV (or streptomycin)	Daily (3 times weekly can be used with aminoglycosides)
	Refractory disease	≥ 4	Macrolide Rifampicin Ethambutol ALIS or amikacin IV	Daily (3 times weekly can be used with aminoglycosides)
*M. kansasii*		3	Macrolide Rifampicin Ethambutol OR	Daily OR 3 times weekly
			Isoniazid Rifampicin Ethambutol	Daily
*M. xenopi*		≥ 3	Macrolide and/or moxifloxacin Rifampicin Ethambutol Amikacin	Daily (3 times weekly can be used with aminoglycosides)
*M. abscessus*		≥ 3	Guided by in vitro susceptibility and in collaboration with experts	Based on expert consultation

ALIS, amikacin liposome inhalation suspension; IV, intravenous; MAC, *Mycobacterium avium* complex. Adapted from [22]

#### Antibiotic delivery via inhalation

As a pulmonary disease, one approach to treating NTM-PD has been to target the lung directly via inhalation. Inhalation of drugs for lung conditions provides precise, direct delivery that can provide high lung concentrations with the potential for reduced systemic exposure and reduced selection pressure for multidrug resistant (MDR) organisms [18, 72].

Most NTM species, particularly MAC, are susceptible to aminoglycoside antibiotics and amikacin has been shown to be an effective concentration-dependent antibiotic against MAC in vitro [64]. Systemic amikacin in multidrug regimens has been associated with higher rates of culture conversion in MAC and *M. abscessus* infections than regimens where amikacin is absent [73, 74] and is recommended as part of current guideline-based therapy (GBT) for those with severe, cavitary or macrolide-resistant MAC-PD [22, 75]. However, systemic administration of amikacin is limited for prolonged use by the emergence of ototoxicity, vestibular toxicity, and renal toxicity, and the correlation between clinical outcomes and MIC is not well established [64]. Similarly, systemic penetration of antibiotics to the lung, including amikacin, is limited [76] requiring increased dosing in order to achieve effective lung concentration [77], which can lead to an increased risk of serious adverse events [78]. Many patients cannot safely reach high enough concentrations for optimal efficacy and are at risk of treatment failure [78]. This presents challenges for an effective drug concentration to combat MAC and *M. abscessus*.

Penetration of some antibiotics, including amikacin, into macrophages and biofilms is low and accumulation in cells such as macrophages is poor [76, 77, 79, 80]. However, for infections like NTM-PD where entry into macrophages and other cells, as well as the formation of biofilms, is common and provides potential reservoirs of infection, penetration of and accumulation in intracellular spaces is essential. Liposomes, as neutral carriers constructed of mammalian membrane-like components, can effectively penetrate both macrophages and biofilms. Liposomes are small, artificial, enclosed spherical vesicles composed of a phospholipid bilayer, which effectively encapsulate hydrophilic molecules or sequester hydrophobic drugs in the lipid bilayer and provide a controlled release system [18]. Liposomes are widely used as drug delivery nanocarriers, with the ability to transport agents to target sites while minimising systemic exposure [18].

Currently, the only treatment specifically developed for the treatment of refractory MAC-PD and approved in the USA, EU and Japan is amikacin liposome inhalation suspension (ALIS) [81]. ALIS is a nebulised liposomal formulation of amikacin which has been specifically designed to meet the three major challenges for MAC-PD: effective antimicrobial activity against MAC; effective and targeted distribution to the point of infection; and effective penetration of intracellular spaces including macrophages and biofilms, where MAC are sequestered [82, 83]. The breakpoint for amikacin resistance for MAC has changed to ≥ 128 μg/mL for liposomal encapsulated formulation due to direct delivery of ALIS to the lung (resistance breakpoint is ≥ 64 μg/mL for IV amikacin) [22, 84, 85] and this should be considered when undertaking amikacin susceptibility testing as recommended by guidelines [22]. ALIS is recommended to be added in adults with MAC-PD who fail to achieve culture conversion after 6 months of oral GBT alone by 2020 international guidelines [22].

ALIS consists of amikacin encapsulated in liposomes composed of dipalmitoylphosphatidylcholine (DPPC) and cholesterol [81, 82]. ALIS is administered using PARI’s Lamira® Nebuliser System, which was optimised for ALIS based on PARI Pharma’s eFlow® nebuliser [82, 83].

Clinical studies demonstrated effective lung penetration of amikacin with ALIS in healthy volunteers and patients with NTM-PD [86, 87]. ALIS also demonstrated effective penetration of macrophages in preclinical studies (in vitro and in vivo animal studies), compared with non-liposomal delivery, along with an ability to penetrate NTM biofilms [88]. In the Phase III randomized controlled clinical study CONVERT, culture conversion was strictly defined as three consecutive monthly negative sputum cultures. ALIS achieved culture conversion in 29% (65 of 224) of patients at month six compared with 9% (10 of 112) treated with oral GBT alone (P < 0.0001), with a serious adverse events rate comparable in both treatment groups (20.2% vs 17.9% at 6 months). Culture conversion was also sustained at 12 months of treatment (18.3% vs 2.7%; P < 0.0001) and durable 3 (16.1% vs 0; P < 0.0001) and 12 months (13.4% vs 0; P < 0.0001) following the end of treatment [89, 90]. ALIS is now being evaluated in newly diagnosed MAC-PD patients in the post-approval studies ARISE and ENCORE (trial registrations: NCT04677543 and NCT04677569).

#### Using existing antibiotics

There has been a long history of managing NTM-PD with antimycobacterial agents typically used for the treatment of TB and leprosy. Clofazimine has been historically used for the treatment of leprosy, but its use has been increasing in the treatment of NTM-PD, despite limited data to support efficacy. Recently, data from various retrospective observational studies have suggested efficacy, supported by a recent meta-analysis which demonstrated a treatment success rate of 56.8% when clofazimine was part of the treatment regimen [91–94]. However, regimens containing clofazimine demonstrated lower rates of treatment success compared with non-clofazimine containing regimens [93]. A Phase II trial is currently underway that will evaluate the efficacy of clofazimine for the treatment of MAC-PD (trial registration: NCT02968212). Novel formulations of clofazimine are currently under investigation for the treatment of NTM-PD, including dry powder inhalation [95], and data are awaited for a new fixed-dose formulation (RHB-204, Redhill Biopharma) of clarithromycin, rifabutin, and clofazimine, which is in a Phase III trial (trial registration: NCT04616924).

Bedaquiline is a diarylquinoline antibiotic indicated for MDR TB. Although less active against NTM compared with *M. tuberculosis*, bedaquiline has demonstrated in vitro bacteriostatic activity against MAC and *M. abscessus*. However, a real-world case series with a limited number of patients (n = 10) suggested that although it was able to improve symptoms and decrease bacterial load, sustained culture conversion after 6 months of treatment was not observed [96]. A Phase II/III trial to evaluate the efficacy and safety of treatment regimens containing bedaquiline in patients with refractory MAC-PD is currently underway (trial registration: NCT04630145).

Antibiotics more commonly used to treat non-mycobacterial infections have also shown some efficacy in NTM-PD. One example is tedizolid, an oxazolidinone typically used to treat acute bacterial skin and skin structure infections (ABSSSI), which has demonstrated efficacy in a macrophage model and in a case study of an immunocompromised patient with *M. abscessus* infection. Omadacycline, also more commonly used for ABSSSI, has similarly demonstrated significant in vitro activity against *M. abscessus*, but clinical data are currently limited to case series [96, 97].

Using previously untried antibiotic combinations in NTM-PD is another approach to repurposing antibiotics; these include vancomycin–clarithromycin for *M. abscessus*-PD. Dual β-lactam combinations have also demonstrated in vitro efficacy against *M. abscessus*-PD in macrophages as well as animal models [98].

#### Novel non-antibiotic therapies and approaches in development

Several novel approaches to the treatment of NTM-PD are being developed without the use of antibiotics. In a prospective pilot study in nine patients with cystic fibrosis who have *M. abscessus* infection, nitric oxide (NO) demonstrated improvements in both forced expiratory volume in one second and six-minute walking distance, and reductions in bacterial load following treatment with inhaled NO [99]. A Phase II proof-of-concept study of inhaled NO in patients with NTM-PD has also been completed (trial registration: NCT03748992) and an open-label study of the at-home NO generator LungFit® GO is currently taking place (trial registration: NCT04685720). In vitro studies have shown potent antibacterial activity against *M. abscessus* following perfusion with NO in combination with clofazimine and amikacin [100]. Further studies are needed to assess the efficacy of NO against *M. abscessus* infection as part of combination therapy, and also its ability to reach bacteria sequestered in biofilms and macrophages.

Another candidate in development is granulocyte–macrophage colony stimulating factor (GM-CSF), which contributes to macrophage activation. Inhalation of GM-CSF may have the potential to enhance the host defence mechanism against *M. abscessus* [96]. A study to explore its utility in *M. abscessus* infection (ENCORE) was terminated in 2021 because of COVID-19 limitations and another (OPTIMA) was completed in 2020, with initial results demonstrating that in patients with severe disease, inhaled GM-CSF did not significantly improve culture conversion rates, although a slight reduction in bacterial load was observed (trial registration: NCT03597347; NCT03421743) [101].

One novel approach to treating MAC and *M. abscessus* infections is that of vaccination. Current data demonstrates that Bacillus Calmette-Guérin (BCG) vaccination-induced immunity exhibits cross-reactivity to MAC and *M. abscessus* and may be effective as a potential prophylaxis or treatment for NTM-PD [102]. In vitro studies have shown that immunity caused by BCG vaccination or latent tuberculosis (TB) infection induces NTM cross-reactive T cells that can inhibit NTM replication within macrophages. In addition, an immune response is elicited when BCG-expanded T cells are exposed to macrophages infected with *M. avium* and *M. abscessus* [102]. Studies in BCG-vaccinated mice and humans have further emphasised these findings that BCG vaccination provides cross-protective immunity against *M. avium* and *M. abscessus* [102]. A Phase II open-label study is currently underway that will assess the role of BCG vaccination in the prevention of infections including those caused by NTM (trial registration: NCT04884308).

Bacteriophage therapy provides another potential novel approach to treat NTM-PD [96, 98], which uses viruses that infect and neutralise infecting bacteria. Although clinical data are currently lacking, a case report of a patient with disseminated *M. abscessus* infection where pulmonary disease predominated demonstrated clearance of infection after receiving treatment with multiple phages [98]. However, a limitation to this therapy includes its poor efficacy against mycobacteria without laboratory manipulation, meaning that practical usage of this method remains far from realised.

Optimising host defences against NTM infection also provides a possible avenue to effective therapy; targeting the inflammatory and immune pathways is currently under exploration. These experimental approaches include enhancing autophagy with mammalian target of rapamycin (mTOR) inhibitors; blocking programmed cell death protein-1 expressed on the surface of macrophages, which may improve host immune defence; and boosting the immune system with interferon-γ (IFN-γ) where in vivo mouse models suggest IFN-γ therapy may enhance the bactericidal capacity of clofazimine [103].

#### Novel antibiotics in development

Several novel antibiotics are also in development for the treatment of NTM-PD. For example, the novel benzimidazole has demonstrated potent bacteriostatic activity in vitro against MAC and *M. kansasii*, with MIC_50_ values ranging from 0.25 to 4 μg/mL for several species of NTM [104, 105]. A Phase IIa study to assess the efficacy and safety of SPR719 for the treatment of *M. avium* complex pulmonary disease (MAC-PD) was put on hold pending discussions with the US Food and Drug Administration, and is due to restart in the second half of 2022 (trial registration: NCT04553406). Two newly developed anti-TB drugs, delamanid and pretomanid, have also been evaluated for activity against *M. abscessus*. Although current data are not encouraging, more in vitro and in vivo data are required to determine their potential for treating *M. abscessus* infections [98].

## Future perspectives

NTM-PD is increasing in prevalence and is a growing public health concern. A better understanding of the microbiology, pathogenesis, and epidemiology is needed to optimise patient care. A recent survey by EMBARC of patient perspectives indicated that development of new, effective drugs with improved tolerability was an imperative [106]. Before recent guideline updates [22], treatment outcomes for NTM-PD were seen to be suboptimal [48], and for patients who failed first-line treatment, options were limited [107]. Development of therapies for NTM-PD requires a focus on overcoming structural barriers of NTM for effective bacterial penetration and penetrating intracellular spaces including phagocytic cells (e.g., macrophages, biofilms) where NTM are sequestered to evade host defences and antimicrobial therapy.

A range of approaches are emerging and are in development to treat NTM-PD that focus mainly on novel antimicrobial therapy but with a view to also capitalise on existing technologies [71, 108]. A key advancement in NTM-PD management was achieved with the approval of ALIS, the first tailored approach for the treatment of refractory MAC-PD in combination with oral GBT. While ALIS is an important therapeutic advance for MAC-PD, both *M. abscessus* and *M. kansasii* remain as challenging pathogens, and a focus to treat these debilitating infections is urgently needed.

## Data Availability

This manuscript does not include data and material that can be shared.

## Abbreviations

ABSSSI: Acute bacterial skin and skin structure infections
ALIS: Amikacin liposome inhalation suspension
BCG: Bacillus Calmette-Guérin
DPPC: Dipalmitoylphosphatidylcholine
GBT: Guideline-based therapy
GM-CSF: Granulocyte–macrophage colony stimulating factor
IFN-γ: Interferon-γ
MAC: *Mycobacterium avium* Complex
MAC-PD: *Mycobacterium avium* Complex pulmonary disease
MDR: Multidrug resistant
MIC: Minimum inhibitory concentration
NO: Nitric oxide
NTM: Non-tuberculous mycobacteria
NTM-PD: Non-tuberculous mycobacterial pulmonary disease
TB: Tuberculosis
